# Unveiling a Novel Molecular Interaction and Pro-Metastatic Signaling Cascades Driven by KRIT1

**DOI:** 10.3390/ijms27083419

**Published:** 2026-04-10

**Authors:** Lucrezia Paradisi, Paolo Guazzi, Matteo Macis, Francesca Finetti, Alfonso Trezza, Raffaella De Paolo, Marta Roncetti, John F. Marshall, Laura Poliseno, Federica Finetti, Lorenza Trabalzini

**Affiliations:** 1 Department of Biotechnology, Chemistry and Pharmacy, University of Siena, Via Aldo Moro 2, 53100 Siena, Italy; lucrezia.paradisi2@unisi.it (L.P.); matteo.macis@student.unisi.it (M.M.); alfonso.trezza2@unisi.it (A.T.); finetti2@unisi.it (F.F.); 2 HansaBioMed Life Sciences Ltd., 12618 Tallinn, Estonia; paolo.guazzi@hansabiomed.eu; 3 Department of Life Science, University of Siena, Via Aldo Moro 2, 53100 Siena, Italy; francesca.finetti@unisi.it; 4 Institute of Clinical Physiology (IFC), Italian National Research Council (CNR), Via Moruzzi 1, 56124 Pisa, Italy; raffaelladepaolo@cnr.it (R.D.P.); roncetti.m@gmail.com (M.R.); laura.poliseno@cnr.it (L.P.); 5 Oncogenomics Unit, Core Research Laboratory (CRL), Institute for the Study, Prevention and Oncology Network (ISPRO), Via Moruzzi 1, 56124 Pisa, Italy; 6 Barts Cancer Institute, Queen Mary University of London, Charterhouse Square, London EC1M 6BQ, UK; j.f.marshall@qmul.ac.uk

**Keywords:** KRIT1, KIF1C, tumor metastasis, cytoskeleton, cancer cell migration, tumor suppressor, molecular modeling, docking simulation

## Abstract

K-Rev Interaction Trapped protein-1 (KRIT1) is a scaffold protein that forms functional protein complexes involved in physiologically important signaling networks. While it is primarily recognized for its association with Cerebral Cavernous Malformations (CCMs), KRIT1 may also play critical roles in tumor formation and the acquisition of malignant phenotypes, regulating cell adhesion, cytoskeletal dynamics, and angiogenesis. In this study, we investigated the role of KRIT1 in cancer cell migration and metastasis, with a focus on identifying novel interacting proteins and characterizing the intracellular signaling pathways activated upon its loss. By using a yeast two-hybrid screening, we identified Kinesin Family Member 1C (KIF1C), a protein involved in regulating podosome and invadopodium elongation, as a novel binding partner of KRIT1, and the interaction was confirmed in melanoma and epithelial cancer cells. In silico docking and interaction interface analyses supported the KRIT1–KIF1C interaction, providing structural insight into the binding mode as shown experimentally. We also found that SRC and focal adhesion kinase (FAK) phosphorylation, as well as Ras homolog family member A (RhoA) expression, represent additional pathways affected by the loss of KRIT1. This study confirms our earlier hypothesis that KRIT1 functions as a tumor suppressor and uncovers a compelling link between its loss and enhanced cancer aggressiveness.

## 1. Introduction

The metastatic cascade is a complex, multi-step process by which cancer cells disseminate from a primary tumor to establish secondary tumors in distant organs. This involves several critical stages, such as degradation and invasion of the surrounding extracellular matrix, intravasation into the bloodstream or lymphatic vessels, survival and transport through the circulatory system, extravasation into distant tissues, and subsequent colonization to form clinically detectable metastases. Metastasis is the leading cause of cancer-related mortality due to its capacity to impair the function of essential organs [1]. Numerous studies have established a strong correlation between distant metastasis in cancer and poor patient prognosis. Elucidating the molecular and cellular mechanisms driving cancer metastasis is crucial for the development of more effective therapeutic strategies and improving clinical outcomes.

The KRIT1 signaling pathway has been studied since mutations in its gene cause Cerebral Cavernous Malformations (CCMs), a rare familial or sporadic vascular disease [2]. KRIT1’s ability to regulate several signaling pathways related to cell–cell interaction, cytoskeleton dynamics, angiogenic processes, oxidative stress, and inflammation has been extensively described [2,3,4,5,6]. Furthermore, KRIT1 has been shown to play an important role in mediating cell motility and migration, not only in endothelial cells but also in non-endothelial cells, such as cancer cells [7] and neutrophils [8]. Despite its main involvement in CCM, few studies have demonstrated that KRIT1 is implicated in other pathologies, including cardiovascular diseases, diabetes [9], intestinal epithelial barrier dysregulation [10], and cancer [7,11,12]. We recently provided the first indications about the role of KRIT1 in the migration and invasion of melanoma cells and melanoma metastasis by using human melanoma specimens [7]. Nevertheless, the molecular functions of KRIT1 remain a research question for understanding the physio-pathological role of KRIT1 during cancer progression.

KRIT1 is a scaffold protein without defined catalytic domains, while it contains several well-defined protein–protein interaction motifs and domains, including a Nudix domain, three Asn-Pro-X-Tyr/Phe (NPx(Y/F)) motifs, a C-terminal Ankyrin Repeat Domain (ARD), and a FERM (band 4.1, ezrin, radixin, moesin) domain [13]. KRIT1 was first identified as a binding partner of the small GTPase RAP1 through a yeast two-hybrid screening (Y2H) [14]. The interaction between KRIT1 and RAP1 occurs via the KRIT1 FERM C-terminal domain, a clover-shaped module constituted by three subdomains (F1, F2, and F3) [15], and regulates fundamental cellular features, such as the stabilization of cell–cell junctions and regulation of intracellular adhesion processes [16,17]. In addition, other molecular interactions mediated by different domains have been described in recent years. It was reported that KRIT1 binds tubulin through the Nudix domain, ICAP1 through the first NPxY motif, SNX17 through the second NPxY motif, CCM2 at the level of the third or second NPxF motif, and finally HEG1 through the FERM domain [13]. We reported that KRIT1 interacts with Nd1-L [18], a cytosolic actin-binding protein involved in the actin cytoskeleton organization [19]. All these interactions drive the KRIT1 cellular signaling, regulating specific signaling information, and control the cellular localization of KRIT1 and of its specific binding proteins. For example, the binding of KRIT1 with RAP1 or with ICAP1α promotes the re-localization of KRIT1 and inhibits the co-sedimentation of KRIT1 with microtubules [20].

Both microtubules and the actin cytoskeleton are highly dynamic and elastic structures that allow a rapid formation of membrane protrusions and play a fundamental role in cell migration. Mesenchymal cell migration involves several phases that depend on actin dynamics (polymerization and depolymerization), cell adhesion, actomyosin contraction cycles, and microtubule reorganization [21,22,23].

To investigate the molecular and cellular mechanisms underlying tumor metastasis, this study evaluates the role of KRIT1 in cancer cell invasion and migration and describes a novel KRIT1 signaling pathway involving the interaction with the kinesin KIF1C.

## 2. Results

### 2.1. KRIT1 Downregulation Is Associated with Cancer Cell Migration and Invasion

We recently demonstrated that KRIT1 silencing in human melanoma cells increases in vitro migration and invasion, through a mechanism linked to the activation of the epithelial–mesenchymal transition (EMT) [7].

**Figure 1:**
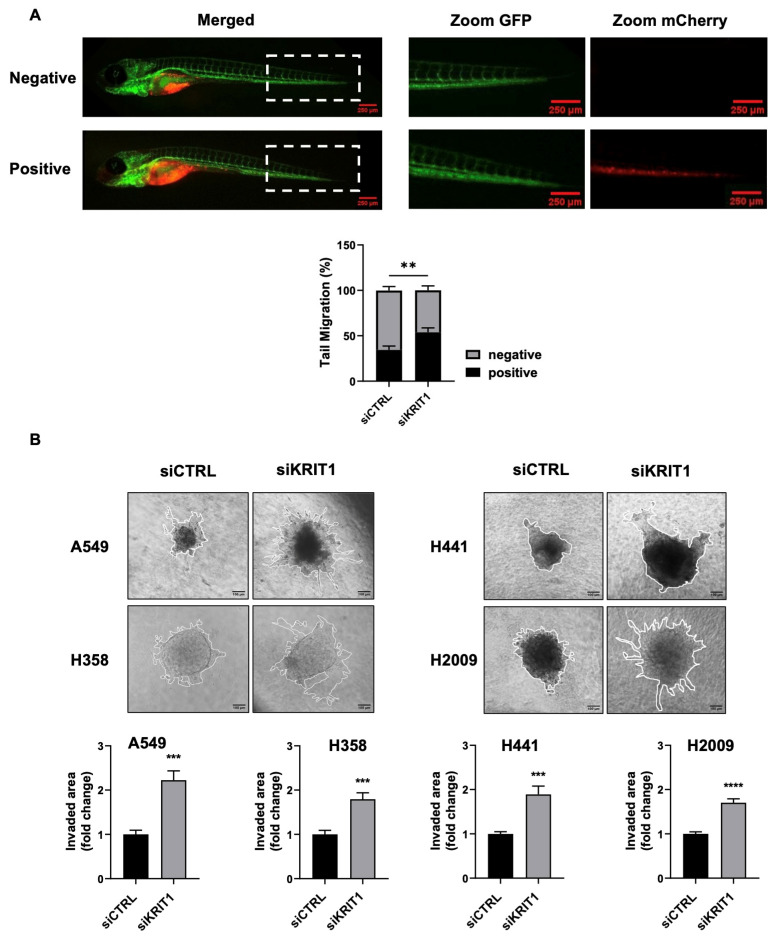

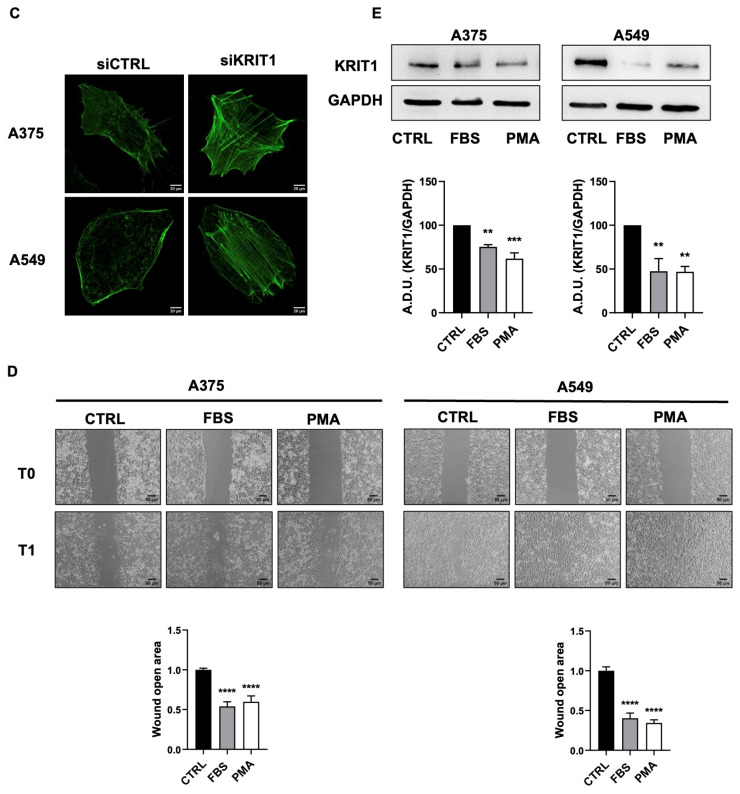
KRIT1 downregulation enhances migratory and invasive properties of cancer cells both in vivo and in vitro. (**A**) A375 transfected with siRNA against KRIT1, or a negative control, and stained red, were injected into the yolk sac of zebrafish embryos. Fish with displaced cells in the tail were considered positive for migration, while those without were negative. Data are expressed as mean ± SEM and are representative of three different experiments with a total of 246 fish for siCTRL and 275 for siKRIT1. ** *p* < 0.01. (**B**) Bright-field images (10×) of spheroids embedded in Matrigel, and collagen matrix composed of KRIT1-knockdown cancer cells and fibroblasts. Data are expressed as mean ± SEM. Images are representative of three independent experiments. Scale bar, 100 µm. *** *p* < 0.001; **** *p* < 0.0001. (**C**) Immunofluorescence analysis of F-actin (phalloidin) in control and KRIT1-knockdown A375 and A549 cells. Representative images (medial optical sections) are shown (*n* = 3). Scale bar, 20 µm. (**D**) Scratch assay of confluent monolayers of A375 and A549, treated with 2% FBS and PMA (80 ng/mL). Images were acquired at T0 and T1 (24 h post-wounding) at 10× magnification. Data are reported as the percentage of wound open area per well and are expressed as mean ± SEM. Results are representative of three independent experiments. Scale bar, 50 µm. **** *p* < 0.0001. (**E**) Western Blotting analysis of A375 and A549 cells treated with 2% FBS and PMA (80 ng/mL) for 48 h. Data are expressed as mean ± SEM and are representative of three independent experiments. A.D.U., arbitrary densitometry units. ** *p* < 0.01; *** *p* < 0.001.

To confirm this observation in in vivo models, we investigated whether KRIT1-silenced A375 melanoma cells could disseminate throughout the zebrafish body. SiCTRL and siKRIT1 A375 cells (Figure S1A) were injected into the yolk sac of 48 h post-fertilization embryos and analyzed at 96 h post-injection using a stereomicroscope. As shown in Figure 1A, we observed a significant spread of cancer cells throughout the tail of the fish in siKRIT1 cells compared to the control. These results strongly support our previous work and push towards more in-depth studies.

To generalize the role of KRIT1 in invasive and migratory phenotypes of cancer cells, we studied these processes in non-melanoma cancer cells by using spheroids formed by lung epithelial tumor cells and fibroblasts in a matrix composed of collagen and Matrigel. Spheroid co-cultures were generated by resuspension of fibroblasts (MRC5) and siCTRL or siKRIT1 cancer cells (A549, H358, H441, and H2009; Figure S1B) in hanging droplets containing methylcellulose. The following day, spheroids were collected and embedded in a 3D matrix in glass-bottomed 96-well plates. Invasion of cells from the center of the spheroid into the surrounding matrix was monitored by light microscopy. As reported in Figure 1B, loss of KRIT1 resulted in an increased ability of epithelial cancer cells to invade into the matrix when co-cultured with fibroblasts.

Accordingly, we also observed that KRIT1 deficiency results in an increased number of stress fibers in both melanoma and epithelial cancer cells (Figure 1C), indicating that the loss of KRIT1 is associated with the acquisition of an invasive phenotype of cancer cells.

In addition, we evaluated the effects of generic chemotactic stimuli such as fetal bovine serum (FBS) and phorbol myristate acetate (PMA) on A375 and A549 cell lines. We discovered that cell migration induced by FBS and PMA (Figure 1D) occurs alongside a reduction in KRIT1 expression (Figure 1E).

These data indicate that KRIT1 loss is associated with increased cancer cell migration and invasion and confers metastatic potential to different types of cancer cells, prompting the search for the molecular mechanisms underlying this effect.

### 2.2. KRIT1 Associates with Motor Proteins Regulating Cell Migration

It is well known that KRIT1 contains domains and motifs known to mediate protein–protein interactions. The presence of these domains, as well as known KRIT1 molecular interactions, strongly indicates that this protein can act as a scaffold for the assembly of functional protein complexes involved in physiologically important signaling networks.

**Figure 2:**
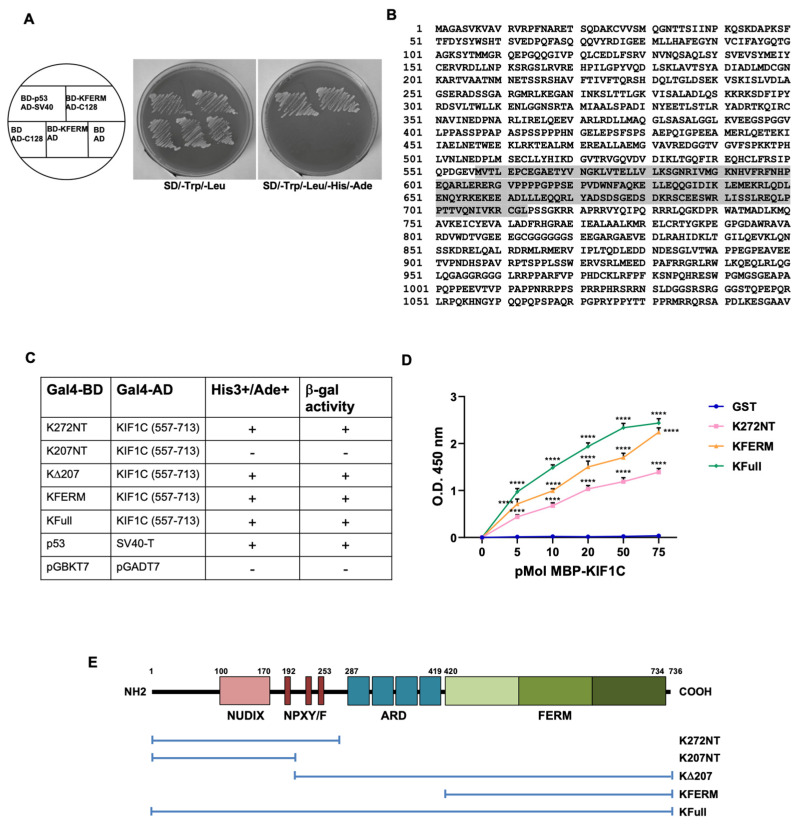
KIF1C is a new interactor of KRIT1. (**A**) Small-scale yeast two-hybrid (Y2H) analysis of the interaction of KRIT1 with the C128 clone. The AH109 yeast strain was cotransformed with GAL4DNA-BD and GAL4AD fusion constructs as indicated (left). Cotransformed AH109 cells were then streaked out on plates lacking Trp and Leu (SD/-Trp/-Leu) or plates lacking Trp, Leu, His and Ade (SD/-Trp/-Leu/-His/-Ade). The interaction between the p53 protein and SV40 large T-antigen was used as a positive control. Empty vectors encoding the GAL4DNA-BD and AD were used as negative control. (**B**) Amino acid sequence of mouse KIF1C. The fragment identified as a KRIT1-FERM interactor in the Y2H screening, KIF1C (557–713), is highlighted in gray. (**C**) Small-scale Y2H analysis of the KRIT1/KIF1C interaction. AH109 cells were cotransformed with the pACT2 vector encoding GAL4AD-KIF1C (557–713) and GAL4DNABD fusion constructs encoding different fragments of KRIT1 and full-length KRIT1. Protein–protein interaction was assayed based on the activation of *HIS3* and *ADE2* reporter genes. Activation of the *LacZ* reporter gene was also assayed by determining the β-galactosidase activity of His+ transformants by using a filter assay. (**D**) Analysis of the KRIT1/KIF1C interaction by an ELISA format binding assay. Binding curves were generated by serially varying the concentration of MBP-KIF1C (556–713) while keeping the amount of GST-K272NT, GST-KFERM, and GST-KFull at 50 pmol/well. Recombinant GST was used as a negative control to determine background binding. Protein–protein interactions were detected with an anti-MBP antibody. Absorbance values are plotted as a function of the test protein concentration. Data are representative of three independent assays performed in triplicate. K272NT: residues 1–272 of KRIT1; KFERM: KRIT1 FERM domain (residues 418–736); KFull: full-length KRIT1. **** *p* < 0.0001. (**E**) Schematic representation of the KRIT1 fragments used in the assays.

To identify new binding partners of KRIT1, we had previously performed a yeast two-hybrid screening of a mouse embryo cDNA library using the GAL4BD-KFERM fusion construct, encoding the FERM domain of KRIT1 fused to the GAL4 DNA-binding domain as bait. From the analysis of 3.5 × 10^7^ independent clones, 34 clones positive by *HIS3*, *ADE2*, *MEL1* and *LACZ* expression, representing potential interaction partners of KRIT1, were initially isolated. The pACT2 plasmids contained in the 34 clones were recovered from the yeast cells and then used in a small-scale Y2H assay to verify the specific interaction with KRIT1. Among the 34 isolated clones, 14 exhibited a KFERM-dependent activation of *HIS3*/*ADE2* genes upon retransformation of yeast cells. These clones were sequenced and compared with known sequences in the GeneBank database. Among the 14 clones, in this work, we focused on clone 128 containing a 468 bp fragment encoding the C-terminal portion (aa 557–713) of the KIF1C protein (GeneID 10749) (Figure 2A,B). KIF1C (Kinesin Family Member 1C) is a member of the kinesin superfamily, a group of motor proteins involved in microtubule-mediated intracellular transport and cell migration [24,25,26,27]. Proteins of this superfamily share a conserved N-terminal domain responsible for ATP and microtubule binding, along with a variable region that mediates interactions with specific partners. The putative interaction between KIF1C and the FERM domain of KRIT1 is of particular interest, in light of several experimental findings supporting an association between KRIT1 and microtubules [28,29]. Moreover, the ability of KIF1C to interact with FERM domain-containing proteins has already been demonstrated by Dorner et al., who isolated KIF1C through a yeast two-hybrid screening using the FERM domain of the PTPD1 protein as bait [30].

To further characterize the newly isolated interaction and map the regions of KRIT1 involved in the binding to KIF1C, we assayed the ability of different regions of KRIT1 to bind the KIF1C fragment by using a small-scale Y2H assay. AH109 cells were cotransformed with the pACT2 vector encoding GAL4AD-KIF1C (557–713) and GAL4DNABD fusion constructs encoding different fragments of KRIT1 and full-length KRIT1 (Figure 2C).

The interaction between the p53 protein and SV40 large T-antigen was used as a positive control. Empty vectors encoding the GAL4DNA-BD and AD (pGBKT7 and pGADT7) were used as a negative control. Protein–protein interactions were assayed on the basis of the activation of *HIS3* and *ADE2* reporter genes (ability of transformants to grow in minimal medium lacking histidine and adenine). Activation of the *LacZ* reporter gene was also assayed by determining the β-galactosidase activity of His+ transformants by using a filter assay. The results of the small-scale Y2H are summarized in Figure 2C. Full-length KRIT1 (KFull) maintains the ability to bind KIF1C in yeast cells. Interestingly, KIF1C is also able to interact with the N-terminal fragment of KRIT1, K272NT. The KRIT1 fragment lacking the first 207 N-terminal amino acids (KΔ207) retains the ability to bind KIF1C, whereas the N-terminal fragment of 207 amino acids (K207NT) does not interact with KIF1C, indicating that the first 207 amino acids of KRIT1 are not sufficient for the interaction.

These results suggest that both the FERM domain and the N-terminal region of KRIT1 are involved in the interaction with KIF1C. In addition, region 207–272 is necessary for the interaction.

To confirm the novel interaction identified by the Y2H analysis, and to perform a quantitative assessment of the interaction between the two different regions of KRIT1 and KIF1C, an in vitro ELISA binding assay was subsequently carried out. The results obtained confirmed that both full-length KRIT1 and the two N- and C-terminal fragments of KRIT1 interact with KIF1C (556–713) but not with the same affinity (Figure 2D). In particular, the extent of binding of the full-length protein (green curve) is greater than that of the partial fragments; furthermore, KFERM (yellow curve) interacts with KIF1C with greater affinity than K272NT (pink curve). These data further support the hypothesis that two distinct regions of KRIT1 are required for the interaction with KIF1C (Figure 2E), as demonstrated by Guazzi et al. for the interaction between KRIT1 and Nd1-L [18].

### 2.3. In Silico Analysis of the KRIT1/KIF1C Interaction

**Figure 3:**
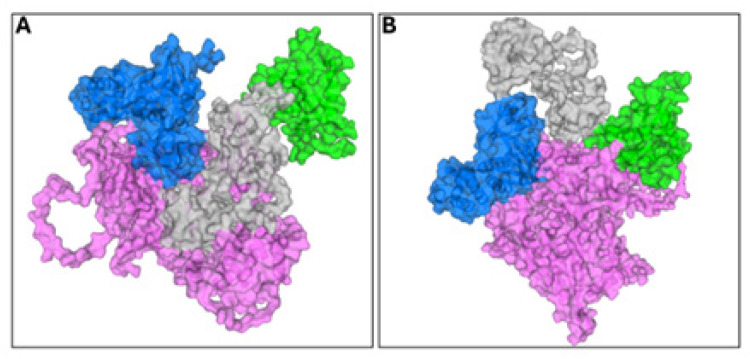
KRIT1/KIF1C docking poses. Two potential interactions were identified: (**A**) KIF1C interacts exclusively with the KRIT1 FERM domain. (**B**) KIF1C interacts with both KRIT1 FERM and N-terminal domains. KRIT1 is represented in green (N-terminal domain), in grey (ankyrin repeat domain), and in blue (FERM domain) surfaces, and KIF1C is represented in pink surfaces.

To further validate the in vitro results, the KRIT1/KIF1C interaction was explored to identify the potential key amino acid residues. GRAMM 2026 software performed and provided the potential KRIT1-KIF1C binding pose. Interestingly, the docking simulation predicted two main docking pose clusters: the first one showed the interaction of KIF1C exclusively on the KRIT1 FERM domain with an energy of interaction of −191.2 kcal/mol (Figure 3A), while the second one exhibited the KIF1C interaction involving both the FERM domain and N-terminal of KRIT1 with an energy interaction of −172.8 kcal/mol (Figure 3B).

Docking analyses yielded comparable interaction energies for binding modes involving either a single KRIT1 region or multiple KRIT1 domains, preventing an unambiguous discrimination based on docking scores alone. Owing to the lack of an experimentally resolved three-dimensional structure of full-length KIF1C and to the low-confidence structural models obtained using AI-based prediction approaches, the binding configuration involving simultaneous contacts with the C- and N-terminal domains of KRIT1 was selected for further analyses, in agreement with previously reported structural evidence [18] and with the interaction patterns observed in the yeast two-hybrid and ELISA assays presented herein (Figure 2E). PDBePISA analysis of the selected complex identified an extended interaction interface encompassing both KRIT1 domains, characterized by a large hydrophobic surface complemented by stabilizing polar interactions (Table 1).

### 2.4. KRIT1 Interacts with KIF1C in Cancer Cells

**Figure 4:**
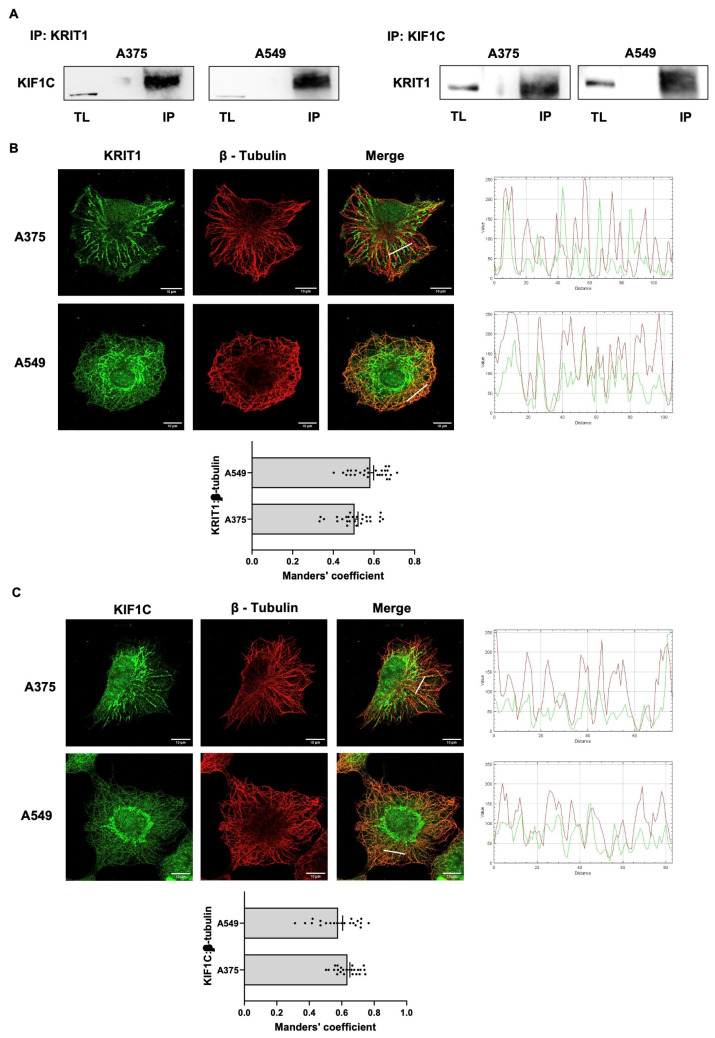

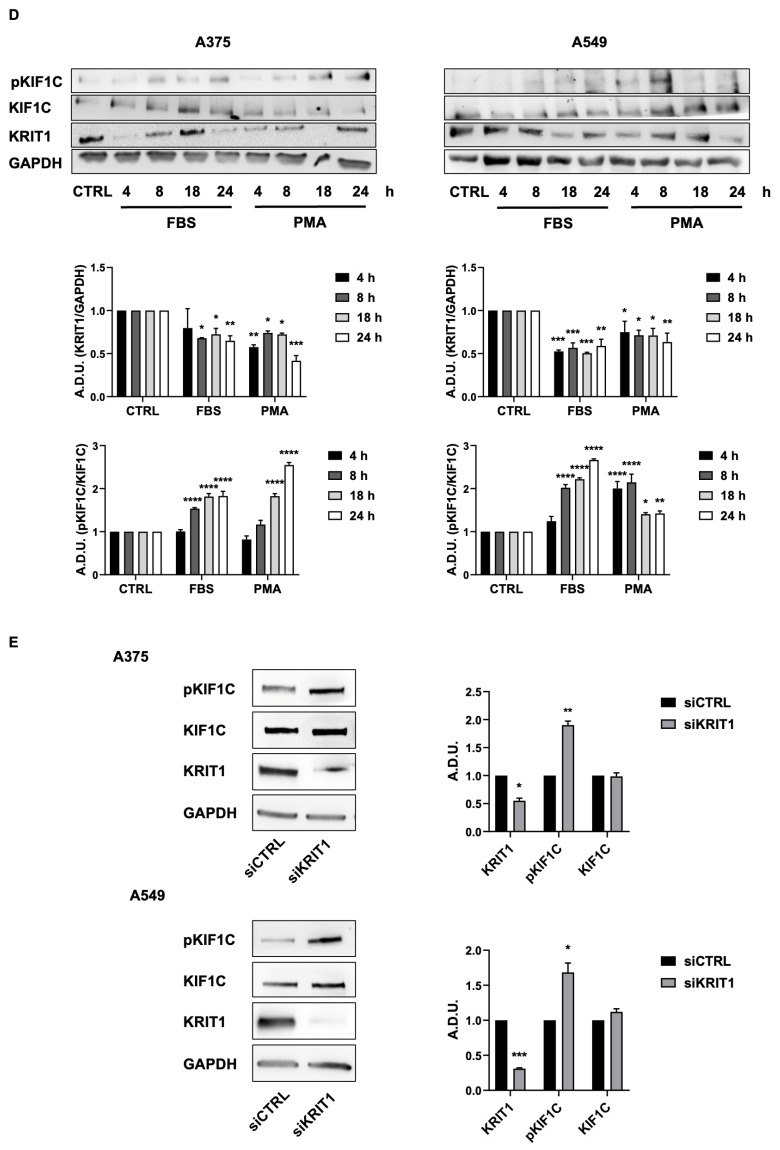
Interaction between KRIT1 and KIF1C in cancer cells. (**A**) A375 and A549 cell lysates were immunoprecipitated with anti-KRIT1 antibody, and samples were immunoblotted using anti-KIF1C antibody (left). Reciprocal immunoprecipitation with anti-KIF1C followed by immunoblotting using anti-KRIT1 antibody was also performed (right). TL, total lysate; IP, co-immunoprecipitate. (**B**,**C**) Immunofluorescence analysis of A375 and A549 cells co-stained for KRIT1 (green) and β-tubulin (red) (**B**) and for KIF1C (green) and β-tubulin (red) (**C**). Intensity profiles along the white lines traced in the overlay images are shown on the right. Quantification (mean ± SEM) of colocalization of KRIT1 (**B**) or KIF1C (**C**) with β-tubulin (10 cells/sample, *n* ≥ 3), performed by calculating Manders’ coefficient, is shown at the bottom. Images are acquired at 63X. Scale bar, 10 µm. (**D**) Western blotting analysis of A375 and A549 cells treated with FBS (2%) and PMA (80 ng/mL) for 4, 8, 18, and 24 h. Data are expressed as mean ± SEM. A.D.U., arbitrary densitometry units. * *p* < 0.05, ** *p* < 0.01, *** *p* < 0.001, **** *p* < 0.0001. (**E**) Western blotting analysis of A375 and A549 cells downregulated for KRIT1. Data are expressed as mean ± SEM and are representative of three independent experiments. A.D.U., arbitrary densitometry units. * *p* < 0.05, ** *p* < 0.01, *** *p* < 0.001.

In order to further confirm the interaction above reported and evaluate the capability of full-length KIF1C to bind KRIT1, we performed co-IP of endogenous KRIT1 and KIF1C in both A375 and A549 cells. As shown in Figure 4A, endogenous KIF1C co-immunoprecipitated in A375 and A549 cell lysates probed with an anti-KRIT1 antibody. KRIT1 was consistently detected when co-immunoprecipitation was performed using the KIF1C antibody (Figure 4A). These data demonstrate that the KRIT1/KIF1C interaction occurs in cancer cells.

Both KRIT1 and KIF1C have been reported to be associated with the cytoskeleton. It has been established that KRIT1 associates with microtubules to form a complex with β-tubulin [28]. It has also been reported that KIF1C binds to non-muscle myosin IIA via its PTPD-binding domain, providing an interface between the actin and tubulin cytoskeletons [31].

Here, to outline the connection between KRIT1 and KIF1C to the cytoskeleton and cellular dynamics, we analyzed the cytoskeletal localization of both proteins. In Figure 4B,C, we showed that not only KRIT1 but also KIF1C colocalized with β-tubulin, indicating a possible interplay between these proteins.

To establish a functional interplay between KRIT1 and KIF1C during cancer cell invasion and migration, we built on the observation that, under promigratory conditions, KIF1C is phosphorylated at invadopodia and supports their continuous elongation [25]. In this context, we measured KIF1C phosphorylation levels following PMA and FBS treatment, which induce cell migration and reduce KRIT1 expression (Figure 1E). As shown in Figure 4D, both treatments are associated with a reduction in KRIT1 expression and a time-dependent increase in KIF1C phosphorylation, underlying a possible functional correlation between KIF1C and KRIT1 in inducing cell migration. To explore this possibility, we analyzed the levels of KIF1C phosphorylation and expression in cancer cells silenced for KRIT1. Figure 4E shows that KRIT1 silencing induced an increase in KIF1C phosphorylation. This data supports the hypothesis that KIF1C represents one of the mediators by which KRIT1 regulates cancer cell migration.

### 2.5. KRIT1 Downregulation Regulates Promigratory Signaling Pathways

It is well known that scaffold proteins regulate several signaling pathways by assembling and coordinating interactions among multiple molecular partners, ensuring specificity and efficiency of signal transduction [32]. Based on these findings, we also evaluated the potential link between KRIT1 and other signaling molecules known to be involved in cell migration. Among these, SRC family kinases have been shown to interconnect various cellular pathways that promote invasion and metastasis, being key regulators of cell morphology and epithelial integrity, primarily through the modulation of actin cytoskeletal dynamics and cellular adhesions. SRC promotes cellular junction disassembly by activating focal adhesion kinase (FAK) [33]. Starting from these observations, we investigated whether KRIT1 downregulation induces SRC and FAK phosphorylation.

**Figure 5:**
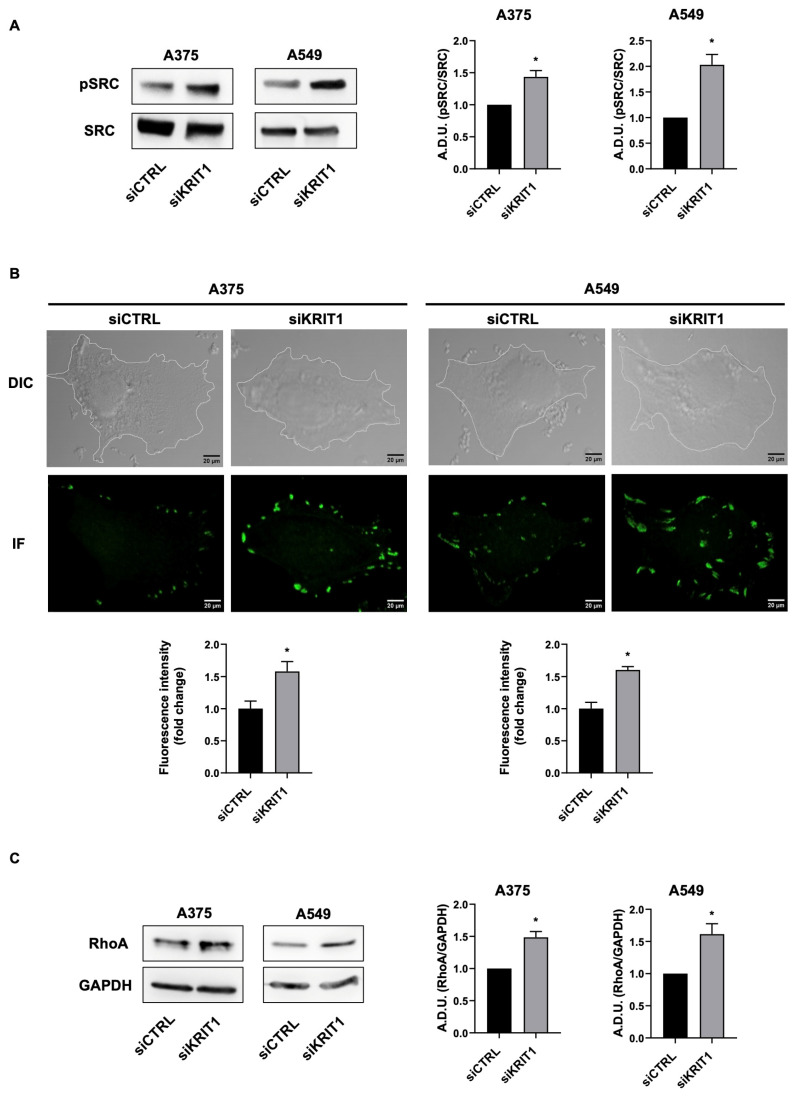
Loss of KRIT1 leads to phosphorylation of SRC and FAK and to increased expression of Rho. (**A**,**C**) Western blotting analysis of SRC phosphorylation and RhoA expression in A375 and A549 cells silenced for KRIT1. Data are expressed as mean ± SEM and are representative of three independent experiments. A.D.U., arbitrary densitometry units. * *p* < 0.05. (**B**) Immunofluorescence analysis of FAK phosphorylation on A375 and A549 cell lines silenced for KRIT1. Representative images (medial optical sections) are shown. The graphs show the quantification of fluorescence intensity. Data are expressed as mean ± SEM. Scale bar, 20 µm. * *p* < 0.05.

As shown in Figure 5A,B, SRC and FAK phosphorylation were increased in cancer cells after KRIT1 silencing, supporting the hypothesis that KRIT1 loss drives cell migration through the activation of several signaling pathways.

To explore this possibility, we also examined the impact of KRIT1 on the Rho family. In fact, the Rho family of small GTPases and ROCK are well-established regulators of actin cytoskeleton organization and dynamics, playing key roles in cell motility and contributing to metastasis [34]. In particular, RhoA is crucial for regulating actin polymerization, membrane bleb formation, turnover of cell–extracellular matrix adhesions at the cell rear, basement membrane disassembly, and cortical contractility [35]. In epithelial cells, KRIT1 has been identified as a negative regulator of RhoA and ROCK [36]. To investigate the effect of KRIT1 on Rho expression in our cellular models, we assessed the expression levels of RhoA after KRIT1 silencing. Figure 5C shows that RhoA expression is increased in the absence of KRIT1. These results confirm that KRIT1 also acts as a negative regulator of RhoA in cancer cells, further supporting its involvement in cytoskeletal dynamics and cell motility.

## 3. Discussion

KRIT1 is a scaffold protein lacking catalytic domains but containing several protein–protein interaction motifs that mediate the formation of functional complexes involved in physiologically important signaling networks [13]. KRIT1 has been extensively studied, as mutations in its gene are associated with Cerebral Cavernous Malformations, a rare vascular disorder that primarily affects the central nervous system. CCMs are characterized by abnormally dilated, leaky capillaries with a thin endothelial lining and a loss of normal structural integrity. KRIT1 regulates multiple signaling pathways involved in cell–cell interactions, cytoskeleton dynamics, angiogenesis, oxidative stress, and inflammation [2]. Moreover, KRIT1 plays a crucial role in cell motility and migration, influencing not only endothelial cells but also non-endothelial cells, including cancer cells [7] and neutrophils [8].

KRIT1 is increasingly recognized as a tumor suppressor gene, with several studies supporting its role in inhibiting cancer progression. In mice with hemizygous KRIT1 deficiency, the loss of a single KRIT1 allele resulted in an increased incidence of small intestinal adenomas and reduced survival compared to controls. This deficiency led to the dissociation of β-catenin from vascular endothelial (VE)-cadherin, its accumulation in the nucleus, and the activation of β-catenin-dependent transcription [16]. In primary tumors, an inverse correlation between KRIT1 and miR-21 expression was observed. miR-21, an oncogenic microRNA overexpressed in tumors, is thought to target KRIT1, which may counteract miR-21’s pro-tumor effects [11]. In melanoma, reduced KRIT1 expression is associated with increased tumor aggressiveness. KRIT1 knockdown led to upregulation of N-cadherin and vimentin, proteins involved in the EMT, thereby contributing to melanoma plasticity. KRIT1 loss also enhances melanoma cell growth, migration, and invasion [7]. In colon cancer, tumor cells regulate KRIT1 expression in endothelial cells via exosomes. Specifically, exosomal miR-21-5p directly suppresses KRIT1 in the endothelium, promoting tumor angiogenesis and vascular permeability, which fuels tumor progression [37]. Furthermore, in prostate cancer cells, PTEN/PI3K/AKT/mTOR-dependent activation of p-PKM2(Y105)/ERα upregulates glyoxalase 1, depleting methylglyoxal-H1 and dampening MG-H1/RAGE signaling. This lowers intracellular H_2_O_2_, reduces KRIT1 expression, and promotes prostate cancer cell proliferation and survival [12].

In this work, we evaluated the role of KRIT1 in two fundamental aspects of cancer metastasis, cell invasion and migration, and we showed that KRIT1 loss is functionally associated with a promigratory and invasive phenotype.

To better understand how KRIT1 may confer migratory properties to cancer cells, we explored the molecular cascade associated with its downregulation.

Firstly, we identified KIF1C as a new KRIT1 molecular interactor, and we investigated the physio-pathological significance of this interaction. The KRIT1/KIF1C complex, initially isolated by Y2H screening, was validated through co-immunoprecipitation of endogenous proteins in human cancer cells. We demonstrated that full-length KRIT1, as well as its N- and C-terminal fragments, bind to KIF1C, albeit with varying affinities. Specifically, full-length KRIT1 exhibited the highest binding affinity, followed by the KRIT1A FERM domain, which showed a stronger interaction with KIF1C than the N-terminal fragment. According to these data, the in silico analysis suggests bimodal interaction and a strong involvement of the FERM domain in the interaction between KRIT1 and KIF1C. Indeed, this domain is implicated in all the binding poses between KRIT1 and KIF1C that were generated.

In depth, the similarity of the interaction energies associated with binding modes involving either a single KRIT1 region or multiple KRIT1 domains did not allow an unambiguous discrimination between the two configurations based solely on docking-derived scores. In addition, the absence of an experimentally resolved three-dimensional structure of full-length KIF1C, together with the inability to generate a reliable structural model using current AI-based prediction approaches due to consistently low confidence and accuracy metrics, precluded the application of molecular dynamics simulations to further dissect and refine the KRIT1–KIF1C complex. Under these methodological constraints, the identification of the most representative KIF1C binding mode on KRIT1 relied on the integration of independent and convergent experimental and computational evidence. Specifically, this choice was guided by the study of Guazzi et al., which demonstrated that the KRIT1/Nd1-L interaction engages both the FERM and N-terminal domains of KRIT1 [18], in conjunction with the binding patterns consistently observed in the yeast two-hybrid and ELISA assays reported herein (Figure 2E). The strong concordance between in silico predictions and in vitro binding data provided a robust rationale for selecting the KIF1C binding configuration simultaneously involving the C- and N-terminal domains of KRIT1 for subsequent analyses. Consistently, structural interrogation of the selected complex using PDBePISA revealed an extensive interaction interface characterized by a large hydrophobic patch complemented by stabilizing polar interactions involving both KRIT1 domains (Table 1), further corroborating the experimentally identified KRIT1 binding region and reinforcing the overall agreement between in silico and experimental observations. To note, the computational modeling was used as a constrained, quantitatively assessed framework to support the experimentally demonstrated direct KRIT1 KIF1C interaction by proposing a thermodynamically plausible interface consistent with the mapped involvement of the KRIT1 FERM region and N-terminal segment, and it provides a rational basis for future structure-guided interface mutagenesis to further refine the residue-level architecture of the complex.

KIF1C is known to be involved in the regulation of cytoskeleton dynamics and rearrangements associated with cell movement and cancer progression, potentially mediating KRIT1 functions in these processes. KIF1C, upon its phosphorylation, enhances its binding to microtubules and promotes invadopodia elongation in cancer cells [25].

In this manuscript, we report that the interaction KRIT1/KIF1C occurs in human cancer cell lines, where both proteins colocalize with β-tubulin, underscoring their association with the cytoskeleton. Furthermore, loss of KRIT1, which has been shown to increase the metastatic potential of cancer cells both in vitro and in vivo, is accompanied by increased KIF1C phosphorylation. Taken together, these findings suggest that KRIT1 and KIF1C cooperate to regulate cancer cell migration.

Second, in this work, we showed that in cancer cells, loss of KRIT1 is also associated with increased activation and/or expression of well-recognized pro-metastatic pathways, such as SRC, FAK, and RhoA.

As is the case for many scaffold proteins, such as IQ motif-containing GTPase-activating protein 1 (IQGAP1) [38], β-arrestins [39] and GIT1/GIT2 [40], which bind numerous interactors involved in the regulation of diverse cellular processes including cytoskeletal organization, transcription, cell adhesion and migration, as well as cell cycle control through multiple signaling pathways, KRIT1 appears to associate with distinct molecular partners, thereby triggering the activation of multiple signaling cascades. As a limitation of this study, we were not able to determine whether the SRC phosphorylation induced by KRIT1 loss could be responsible for KIF1C phosphorylation, as previously reported [25]. Future and in-depth analysis will be conducted to verify this hypothesis.

These results underscore the pivotal role of KRIT1, in cooperation with its binding partners, in regulating cytoskeletal dynamics and cell motility. In addition, we demonstrate that KRIT1 may regulate the metastatic process in both melanoma and epithelial cell lines, supporting the hypothesis that KRIT1 has broader relevance in tumor biology. Future research should focus on validating this mechanism in vivo; however, preliminary data obtained from a limited number of melanoma samples [7] already support the in vitro findings.

## 4. Materials and Methods

### 4.1. Plasmids and Fusion Proteins

The following constructs for Y2H experiments were prepared in a previous work [18]: pGBKT7-KFull, containing full-length KRIT1 (KFull); pGBKT7-KFERM, containing the FERM domain of KRIT1 (418–736) (KFERM); pGBKT7-K207NT (KRIT1 1–207 residues); pGBKT7-K272NT (KRIT1 1-272 residues); and pGBKT-KD207 (KRIT1 208–736 residues). The expression of GAL4BD and GAL4AD fusion proteins was verified by Western blotting analysis of transformed yeast protein extracts with anti-GAL4-DNA-BD or anti-HA epitope mAbs, respectively.

The expression constructs encoding GST-tagged KFull, KFERM, and K272NT were prepared in a previous work by cloning the different inserts into the pGEX-4T1 vector [18].

To prepare the expression construct encoding MBP-tagged KIF1C (557–713), KIF1C (557–713) was obtained by enzymatic digestion of the pACT2 construct isolated by Y2H screening and cloned as an EcoRI/BamHI insert into the pMAL-c2X vector (New England Biolabs, Ipswich, MA, USA). The constructed plasmid was verified by sequencing.

The different GST-tagged proteins were expressed and purified by using the GST Gene Fusion System (GE Healthcare, Milano, Italy). Induction was performed by incubating transformed *E. coli* BL21 cells at 18–28 °C with IPTG (0.1–0.2 mM). GST fusion proteins were finally analyzed by Western blotting with anti-GST antibody (Amersham Bioscience, Little Chalfont, UK). The amount of expressed proteins was determined by using a BCA protein assay kit (Thermo Fisher Scientific, Waltham, MA, USA).

The MBP-tagged protein was expressed and purified by using the pMAL Protein Fusion and Purification System (New England BioLabs). Induction was performed by incubating transformed *E. coli* TB1 cells at 37 °C with IPTG (0.5 mM). MBP fusion proteins were finally analyzed by Western blotting with anti-MBP antibody (New England Biolabs). The amount of expressed proteins was determined by using a BCA protein assay kit.

### 4.2. Yeast Two-Hybrid Analysis

Y2H experiments were performed using the GAL4-based MATCHMAKER Two-hybrid system 3 (Clontech Laboratories, Palo Alto, CA, USA).

For initial screening, the KFERM fragment fused with GAL4BD in the pGBKT7 vector was used as bait and transformed into the AH109 (MATa) yeast strain, which was then mated with the Y187 (MATa) yeast strain pre-transformed with a mouse embryo cDNA library fused with GAL4AD in the pACT2 vector. Diploid cells, containing the reporter genes *HIS3*, *ADE2*, *MEL1*, and *LACZ*, were plated on selective medium. The clones resulting positive by the 4 reporter genes’ expression were candidates for harboring interacting hybrid proteins. To verify the specific interaction between bait and prey proteins, the pACT constructs carrying potential positive-interacting cDNAs were rescued from yeast cells and reintroduced into AH109 cells pre-transformed with pGBKT7-K1AFERM- or pGBKT7. Those cDNAs that exhibited a KFERM-dependent *HIS3/ADE2*-positive genotype upon retransformation were sequenced by using a GAL4AD sequencing primer and compared with known sequences in GeneBank. The sequence of clone C128, studied in this work, was aligned with the nucleotide sequence NM_001039512.

For small-scale assays, AH109 cells were cotransformed with pGBKT7 and pGADT7 constructs; cotransformants were then selected on minimal medium lacking Trp and Leu. Protein–protein interactions were assayed based on the ability of transformants to activate *HIS3*, *ADE2*, and *LACZ* reporter genes. AH109 cells cotransformed with pGBKT7-53, encoding a GAL4DNA-BD fusion with the murine p53 protein, and pTD1, encoding a GAL4AD fusion with the SV40 large T antigen, were used as positive controls, whereas yeast cells cotransformed with empty pGBKT7 and pGADT7 vectors were used as negative controls. To evaluate the β-galactosidase activity of transformants, a colony lift filter assay was performed as previously described by following the manufacturer’s instructions (Clontech) [18].

### 4.3. ELISA Format Binding Assay

An amount of 100 µL of “coating buffer” containing approximately 50 pmol of the three fragments GST-KFull, GST-K272NT, and GST-KFERMA or GST was added to the wells of an ELISA plate (Nalgene Nunc International, NY, USA). After incubation at 4 °C for 16 h, the wells were washed twice with 100 µL of 0.1% BSA in PBS (*w*/*v*) (Sigma-Aldrich, St. Louis, MO, USA). Each well was incubated for 2 h at 37 °C with 100 µL of 0.1% BSA in PBS (*w*/*v*). At the end of the incubation, each well was washed twice with 100 µL of the same buffer used for saturation. Variable amounts (5–75 pmol) of MBP-KIF1C in 100 µL 0.1% BSA in PBS (*w*/*v*) were added to the wells. After incubation for 1 h at 37 °C, the wells were washed three times with 100 µL of 0.1% Tween-20 in PBS (*v*/*v*). An amount of 100 µL of anti-MPB diluted 1:1000 in PBS was added to each well. Incubation was performed for 1 h at room temperature. The wells were then washed three times with 100 µL 0.1% Tween-20 in PBS (*v*/*v*). To each well, 100 µL of peroxidase-conjugated anti-rabbit IgG (Sigma-Aldrich, MO, USA) diluted 1:30.000 in PBS was added. After incubation for 1 h at 37 °C, the wells were washed three times with 100 µL of 0.1% Tween-20 in PBS (*v*/*v*) and then spiked with 200 µL of TMB. The reaction was stopped after approximately 30 min by the addition of 100 µL of 0.5 M H_2_SO_4_.

The intensity of the staining in each well was determined by measuring the absorbance at 450 nm in an ELISA plate reader (EnVision, PerkinElmer Italia, Milano, Italy). Statistical analysis was performed with ANOVA.

### 4.4. In Silico Analysis of KRIT1/KIF1C Interaction

The KRIT1 and KIF1C primary structures were retrieved from the UniProtKB reviewed (Swiss-Prot) database [41] with Entry code O00522 and O43896, respectively.

The KRIT1 and KIF1C primary structures were used as target sequences, and their 3D structures were generated through an AI algorithm named AlphaFold 3 [42]. AlphaFold predicted 5 different 3D structures for each system using different weights and ranked them from best to worst by their mean predicted Local Distance Difference Test (pLLDT) (0–1, higher is better). We selected the first model as the best 3D structure generated by AlphaFold, showing mean pLDDT and uncertainty values of 83.72% and 3.5% for KRIT1 and 66.29% and 7.35% for KIF1C, suggesting a good overall quality of the 3D structures obtained; however, other protein regions were unmodeled with a very low quality score.

To further optimize and relax the 3D structures for the docking simulation, molecular modeling and energy minimization were performed using PyMOD3.0 with MODELLER 10.5 [43], using as a template and target sequences the 3D structures generated by AlphaFold and the primary structures of KRIT1 and KIF1C, respectively. Lastly, the 3D structures were validated through PROCHECK analyses [43]. The CHARMM-GUI platform [44] was used to assign all molecular parameters for the energy minimization using charmm36-mar2019 force field, while GROMACS 2019.3 [45] with CUDA Support was used to perform an energy minimization as suggested in a previous work [46]. Briefly, the structure was immersed in a cubic box filled with TIP3P water molecules and counter ions to neutralize the net charge of the system. The simulation was run applying periodic boundary conditions. The energy of the system was minimized with 5.000 steps using the steepest descent algorithm to converge to a minimum energy with forces less than 100 kJ/mol/nm. The equilibration was performed integrating each time step of 2 fs; a V-rescale thermostat maintained the temperature at 300 K, and a Noose–Hoover barostat maintained the system pressure at 1 atm, with a low dumping of 1 ps^−1^. The LINCS algorithm constrained the bond lengths involving hydrogen atoms. The 3D structures of KRIT1 and KIF1C were extracted and used as starting structures for the docking simulation.

Protein–protein interaction between KRIT1 and KIF1C was predicted by the GRAMMX 2026 tool [47]. In detail, free docking was chosen as the docking methodology, selecting the number of 10 top matches to output as PDB files. The docking parameters were set choosing 60,000 as number of scans matches to output, enabling the clustering of docking poses. The number of top predictions to perform clustering and the clustering threshold (RMSD < A) were set to 60,000 and 6A, respectively. Based on a previous work, we added in the “interface residue constraints” section the KRIT1 FERM domain residues (420–734) as “active” residues (potentially involved in the interaction); all other residues were defined as “passive” (potentially not involved in the interaction).

The first binding pose was selected as best docking pose, and the PDBePISA 2026 tool [48] revealed the binding residues mostly involved in polar interactions between KRIT1 (458, 459, 462, 513, 631, 632, 684, 685, 698, 703, 723, 724, 731, 732, 734, 735, and 736) and KIF1C (21, 22, 24, 254, 257, 258, 259, 264, 504, 505, 506, 527, 638, 640, 641, 643, 644, 651, 652, 658, and 740); thus, a rational docking was performed using the same parameters previously described, adding as “active” residues the residues involved in the polar interaction network.

### 4.5. Cell Culture

HEK293 human embryonic kidney cells, A375 malignant melanoma cells, and A549 lung carcinoma cells (ATCC, VA, USA) were cultured in DMEM (Euroclone, Pero (MI), Italy). H2009 lung adenocarcinoma cells (ATCC) were cultured in DMEM F12 (Gibco, Grand Island, NY, USA, Thermo Fischer Scientific). MRC5 lung fibroblast cells (ATCC) were cultured in EMEM (Sigma-Aldrich). H358 bronchioalveolar carcinoma cells and H441 lung papillary adenocarcinoma cells (ATCC) were cultured in RPMI 1640 (Gibco). All the media were supplemented with 10% fetal bovine serum (Euroclone), 100 U/mL penicillin–streptomycin (Euroclone), and 4 mM L-glutamine (Euroclone). The H2009 medium was also supplemented with 0.005 mg/mL insulin, 0.01 mg/mL transferrin, 30 nM sodium selenite, 10 nM hydrocortisone (Invitrogen, Carlsbad, CA, USA, Thermo Fischer Scientific), 10 nM β-estradiol (Sigma-Aldrich), extra 2 mM L-glutamine, and 5% fetal bovine serum. The cells were grown at 37 °C and 5% CO_2_.

### 4.6. Co-Immunoprecipitation Experiments

A375 and A549 cells were seeded (1.0 × 10^6^ cells/well) in a 100 mm Petri dish with DMEM with 10% FBS. After 48 h, cells were lysed and centrifuged at 13,000× *g* for 15 min at 4 °C. The protein content was measured using a BCA protein assay kit. For co-immunoprecipitation experiments, aliquots of cell extract supernatants containing an equal amount of proteins (100 μg) were combined with 25 μL (0.25 mg) of magnetic beads (Thermo Scientific). The reaction volume was adjusted to 150 µL with Ripa Buffer (Cell Signaling Technology, Beverly, MA, USA). The reaction was maintained for 1 h at 4 °C with mixing. After, the supernatant was combined with 1 µg of anti-KRIT1 or anti-KIF1C antibody (Abcam, Cambridge, UK) and incubated overnight at 4 °C with mixing. The day after, the antigen–sample mixture was added with 25 μL (0.25 mg) of pre-cleaned magnetic beads and incubated at room temperature for 1 h with mixing. After washing, 25 µL of 0.1 M glycine, pH 2.0, was added and incubated for 10 min at room temperature with mixing. The beads were magnetically separated, and the supernatant containing the target antigen was recovered. An amount of 25 µL of neutralization buffer, Tris 1 M pH 7.5–9, and Laemmli buffer were added to perform Western blot analysis.

### 4.7. Immunofluorescence Experiments

A375 and A549 cells were seeded (5.0 × 10^4^ cells/well) on glass coverslips. After 24 h, cells were fixed with cold acetone for 5 min on ice. Cells were then incubated with 3% BSA for 1 h and stained overnight at 4 °C with primary antibody for KRIT1 (1:100, Abcam), KIF1C (1:300, Abcam), β-Tubulin (1:300, Santa Cruz Technologies, Dallas, TX, USA), or pFAK (1:200, Cell Signaling Technology). Slips were washed three times with 0.5% BSA and then incubated for 1 h at room temperature with secondary antibodies Alexa Fluor Rabbit 488 (1:500, Invitrogen), Alexa Fluor Mouse 555 (1:500, Cell Signaling Technology), Alexa Fluor Mouse 488 (1:2000, Invitrogen), or Alexa Fluor Rabbit 555 (1:1000, Cell Signaling Technology). Alternatively, slips were incubated with Phalloidin-488 for 15 min (1:20, Cell Signaling Technology). Confocal microscopy was carried out on a Zeiss LSM700 (Carl Zeiss, Jena, Germany) using a 63X objective. Detectors were set to detect an optimal signal below the saturation limits. Images were processed with Zen 2009 image software (Carl Zeiss, Jena, Germany). Colocalization analyses were carried out on median optical sections using ImageJ 1.53 (2022) and the JACoP plugin to calculate Manders’ coefficient M1 (mean of values obtained in three different regions for each cell). This coefficient indicates the proportion of the green signal coincident with red signal. Manders’ coefficients range from 0 to 1, corresponding to non-overlapping images and 100% colocalization between both images, respectively. Statistical analysis was performed with a Mann–Whitney test.

### 4.8. siRNA Transfection

A375 and A549 cells were plated (2.5 × 10^5^ cells/well) in a 6-well multiplate and, after adhesion, transfected with 10 nM of negative control or KRIT1 siRNA (Qiagen, Hilden, Germany) using 5 µL of lipofectamine 3000 (Invitrogen) according to the manufacturer’s instructions. At 24 h post-transfection, the media were changed. Cells were lysed 72 h post-transfection and further analyzed by Western blotting.

### 4.9. Scratch Assay

A375 and A549 cells were seeded (3.0 × 10^5^ cells/well) in a 24-well multiplate in DMEM 10% FBS. After 24 h, cell monolayers were scored vertically down the middle of each well with a sterile tip. Each well was washed with PBS to remove detached cells. Cells were treated with 2% FBS and with PMA (ChemCruz, Singapore) at a final concentration of 80 ng/mL. Images of the wound in each well were acquired at time 0 and after 24 h with 10× magnification (Eclipse Ts2, Nikon Corporation, Tokyo, Japan). Images were analyzed with ImageJ 1.53 (2022), and results were expressed as arbitrary units of wound and percentage of healing, taking as reference the area at time 0. Statistical analysis was performed with ANOVA.

### 4.10. Hanging Drop Spheroid

H358, H441, H2009, and A549 cells were seeded (2.5 × 10^5^ cell/well) in a 6-well multiplate in the corresponding medium with 10% FBS. After 24 h, cells were transfected with the siRNA. After another 24 h, hanging droplets were seeded. Each droplet was 20 µL and consisted of methylcellulose and a cell volume containing 4.4 × 10^4^ cancer cells (H358, H441, H200) plus 2.2 × 10^4^ fibroblast cells (MRC5) or 2.2 × 10^4^ cancer cells (A549) plus 4.4 × 10^4^ fibroblast cells (MRC5). The day after, spheroids were harvested in the organotypic gels made by high-concentration collagen (final concentration 2 mg/mL) (Corning, New York, NY, USA), Matrigel (175 µL) (Corning), Hepes (1 M, pH 7.4, 25 µL), NaOH (1 N, 10 µL), and media up to 1 mL.

Images of each spheroid were acquired after 5 (H2009), 7 (A549) or 8 days (H358 and H441) after siRNA treatment with 10× magnification (Zeiss, Axiovert 135). Images were analyzed using ImageJ and statistics were performed using a T-Test.

### 4.11. Xenograft in Zebrafish Embryos

A375 cells (1.5 × 10^5^ cell/well) were transfected with siRNA. After 48 h, cells were harvested using trypsin, counted, centrifuged (5 min, 300× *g*) and washed once with PBS. Then, cells were resuspended in 5 mL of PBS solution containing 5 µL of C7001 Cell Dye 1 mg/mL (Invitrogen) and incubated for 15 min at 37 °C and then 15 min at 4 °C. Stained cells were centrifuged again (5 min, 300× *g*) and resuspended in 1 mL of PBS. Cell suspensions were transferred to a 1.5 mL tube and subjected to an additional centrifugation step (5 min, 300× *g*). PBS was completely removed, and the tube was cooled down on ice. Cells were washed once in FBS and then in PBS. Cells were finally resuspended with PBS at a final concentration of 5 µL per 1 × 10^6^ cells.

Concurrently with cell staining procedures, 48 hpf (hours post fertilization) zebrafish embryos of the Casper and the Tg (kdrl: EGFP) were dechorionated manually by forceps (Dumont No. 5, #F6521-1EA, Sigma-Aldrich) and anesthetized with 0.17 mg/mL tricaine (Sigma-Aldrich, A5040). Cell suspension was loaded into a borosilicate glass capillary, and 1 nL (200 cells for migration) was injected into the yolk sac of the dechorionated embryos, using a microinjector (Tritech Research, Los Angeles, CA, USA). Then, embryos were incubated at 36 °C for 96 h. At least 80 embryos were injected per experimental condition, and each experiment was repeated three times. At the end of the treatment period, fluorescence imaging was carried out using a Leica MZ10F Stereomicroscope equipped with a DFC3000 G camera and Leica Application Suite X. Pictures were taken after microinjection and after 96 h of treatment. The migration was positive if the cancer cells reached the tail of the fish and negative if they remained confined to the yolk sac. Data were analyzed using the Fisher test.

### 4.12. Sample Preparation for Western Blotting Analysis

A375 and A549 cells were seeded (2.5 × 10^5^) in 60 mm Petri dishes and grown at 37 °C and 5% CO_2_. After 24 h, cells were starved for another 24 h with fresh medium 0.1% FBS. Cells were then treated with 2% FBS and 80 ng/mL PMA and lysed 4, 8, 18, 24, and 48 h post-treatment. Cell lysates were further analyzed by Western blotting.

### 4.13. Western Blotting Analysis

Cell lysates were centrifuged at 13,000× *g* for 15 min at 4 °C. Protein content was measured using a BCA protein assay kit. For Western blotting analysis, aliquots of cell extract supernatants containing an equal number of proteins (50 μg) were treated with Laemmli buffer (Bio-Rad, Hercules, CA, USA), boiled for 5 min, resolved on a 4–20% stain-free gel (Bio-Rad), and then blotted onto a nitrocellulose membrane (Bio-Rad). Membranes were incubated overnight with the following primary antibodies: anti-KRIT1 (1:1000, Abcam), anti-KIF1C (1:1000, Abcam), anti-phospho-KIF1C (1:1000, Invitrogen), anti-RhoA (1:1000, Cell Signaling Technology), anti-pSRC (1:1000, Cell Signaling Technology), anti-SRC (1:1000, Cell Signaling Technology) or anti-GAPDH (1:1000, Cell Signaling Technology). The membranes were then incubated with 1:3000 dilutions of horseradish peroxidase-conjugated secondary antibody (Cell Signaling Technology) for 1 h at RT. Chemiluminescence was detected by an Image Quant Las 4000 imager (GE Healthcare). Data were analyzed with ImageJ software, and statistical analysis was performed with a T-Test or ANOVA. Results were expressed as arbitrary densitometry units (A.D.U.s).

## 5. Conclusions

In the present work, we investigated the role of KRIT1 in two fundamental aspects of cancer metastasis, namely cell invasion and migration. Specifically, we demonstrated through multiple experimental analyses that the loss of KRIT1 is associated with a migratory and invasive phenotype, and we proposed several signaling pathways activated following KRIT1 loss that contribute to cancer cell migration and invasion.

Firstly, we identified KIF1C as a novel binding partner of KRIT1. KIF1C is known to regulate cytoskeletal dynamics and rearrangements associated with cell movement and cancer progression, and we demonstrated that KRIT1 and KIF1C colocalize with β-tubulin. Moreover, KRIT1 loss was shown to increase KIF1C phosphorylation. Secondly, KRIT1 loss was associated with SRC and FAK phosphorylation, as well as increased RhoA expression. These findings highlight the central role of KRIT1, in cooperation with different binding partners, in regulating cytoskeletal dynamics and cell motility.

Taken together, the data presented in this study extend our understanding of the molecular mechanisms underlying the anti-cancer activities of KRIT1. They also suggest a potential molecular pathway involved in the acquisition of an aggressive phenotype, providing a scientific basis for further diagnostic and therapeutic strategies and for the identification of new potential targets for cancer treatment.

## Figures and Tables

**Table 1:** KRIT1 and KIF1C binding residues. In round brackets are reported the atom types (HB-SB-donor/acceptor) of residues involved in H-bond and Salt Bridge interactions.

KRIT1 (Binding Residue)	KIF1C (Binding Residue)	Distance (Å)	Bond Type
Arg- 29 (NH2)	Ile-539 (O)	3.75	H-bond
Arg-734 (NH1)	Glu-506 (OE2)	3.85	H-bond
Arg-734 (O)	Asn-505 (ND2)	2.92	H-bond
Asn-735 (N)	Asn-505 (OD1)	3.74	H-bond
Asp-137 (OD1)	Gln-542 (NE2)	3.54	H-bond
Cys-685 (N)	Ser-257 (O)	2.99	H-bond
Gln-136 (OE1)	Gln-542 (N)	3.65	H-bond
Gln-458 (NE2)	Asp-638 (OD2)	3.83	H-bond
Gln-459 (OE1)	Leu-641 (N)	3.40	H-bond
Gln-689 (OE1)	Lys-658 (NZ)	2.84	H-bond
Glu-223 (O)	Asn-652 (ND2)	3.46	H-bond
Glu-631 (N)	Ser-21 (O)	2.95	H-bond
Glu-631 (O)	Ala-24 (N)	3.85	H-bond
Glu-703 (O)	Arg-254 (NH1)	3.16	H-bond
Gly-684 (O)	Gly-259 (N)	2.81	H-bond
Gly-684 (O)	Ser-258 (N)	3.13	H-bond
Ile-632 (N)	Gln-22 (O)	2.63	H-bond
Lys- 31 (NZ)	Gln-527 (OE1)	3.39	H-bond
Lys-724 (NZ)	Met-643 (O)	3.84	H-bond
Met-723 (N)	Glu-651 (OE1)	3.67	H-bond
Pro-731 (O)	Arg-264 (NH1)	3.67	H-bond
Ser-736 (OG)	Leu-504 (N)	3.66	H-bond
Thr-20 (O)	Arg-740 (NH1)	2.54	H-bond
Thr-462 (OG1)	Glu-644 (OE1)	3.54	H-bond
Thr-462 (OG1)	Lys-640 (NZ)	3.36	H-bond
Thr-732 (OG1)	Arg-264 (NH2)	2.48	H-bond
Arg-513 (NE)	Glu-644 (OE2)	3.97	Salt Bridge
Arg-513 (NE)	Glu-644 (OE1)	3.03	Salt Bridge
Arg-513 (NH1)	Glu-644 (OE1)	3.44	Salt Bridge
Arg-513 (NH2)	Glu-644 (OE2)	2.85	Salt Bridge
Arg-734 (NE)	Glu-506 (OE2)	3.64	Salt Bridge
Arg-734 (NH1)	Glu-506 (OE2)	3.85	Salt Bridge
Lys-31 (NZ)	Glu-541 (OE1)	3.00	Salt Bridge

## Data Availability

The original contributions presented in the study are included in the article; further inquiries can be directed to the corresponding author.

## Supplementary Materials

The following supporting information can be downloaded at https://www.mdpi.com/article/10.3390/ijms27083419/s1.

## Abbreviations

The following abbreviations are used in this manuscript:

CCM	Cerebral Cavernous Malformations
EMT	Epithelial–mesenchymal transition
FAK	Focal adhesion kinase
FBS	Fetal bovine serum
GRAMM	Global Range Molecular Matching
KIF1C	Kinesin Family Member 1C
KRIT1	K-Rev Interaction Trapped protein-1
PDBePISA	Proteins, Interfaces, Structures and Assemblies
PMA	Phorbol myristate acetate
RhoA	Ras homolog family member A
