# Supplementary figures and images for: Unveiling a Novel Molecular Interaction and Pro-Metastatic Signaling Cascades Driven by KRIT1
Source: Int J Mol Sci. 2026 Apr 10;27(8):3419. doi: 10.3390/ijms27083419 (PMC13117016; doi:10.3390/ijms27083419)

Figure S1

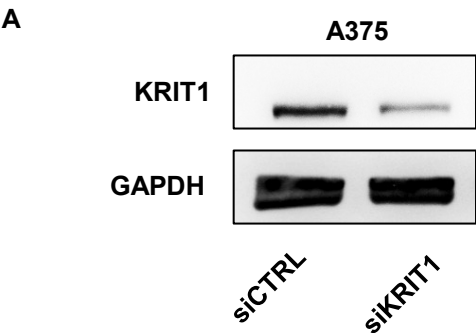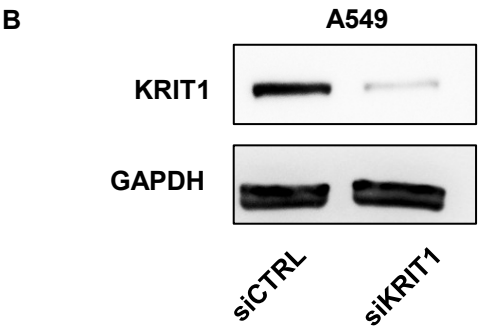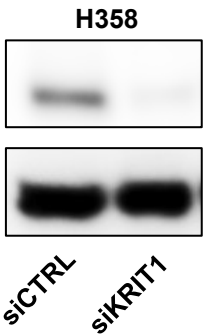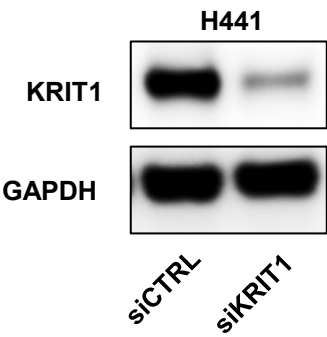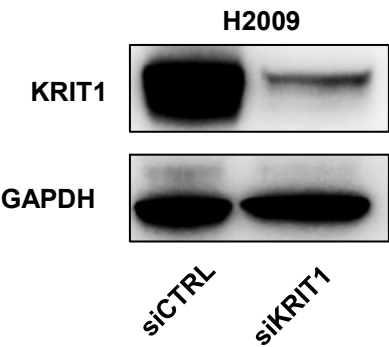

Supplement: Supplementary file 1 [file ijms-27-03419-s001.zip › ijms-4185710-supplementary.pdf]
